# Translational research in Huntington’s disease: opening up for disease modifying treatment

**DOI:** 10.1186/2047-9158-2-2

**Published:** 2013-01-25

**Authors:** Jean-Marc Burgunder

**Affiliations:** 1 Swiss Huntington’s Disease Centre, Department of Neurology, University of Bern, Neurobu Clinics, Steinerstrasse 45, CH 3006, Bern, Switzerland; 2 Departments of Neurology, West China Hospital, Sichuan University Chengdu, Xiangya Hospital, Central South University, and Changsha, Sun Yat Sen University, Guangzhou, China

## Abstract

Research on the molecular mechanisms involved in Huntington’s disease, a monogenic disorder with a complex phenotype including motor, behaviour, and cognitive impairments, is advancing at a rapid path. Knowledge on several of the multimodal pathways has now lead to the establishment of rational strategies to prepare trials of several compounds in affected people. Furthermore, improved understanding of the phenotype and on ways of assessing it, as well as the process of developing biomarkers, allows setting the frame for such studies. In this brief review, the present status of some of these aspects is examined.

## Review

Huntington’s disease is a complex autosomal-dominant disorder with a variable phenotype comprising motor, cognitive and psychiatric symptoms and signs [1,2]. The mutation is fully penetrant and leads to death after a relentless progressive course of impairment with a gradual loss of function until death after 15–25 years. HD is a monogenetic degenerative disorder, which can be diagnosed well in advance of any symptom, opening up opportunities for a disease-modifying treatment long before symptom onset, which typically occur around 40 years old. This disorder may therefore be considered as a paradigm for the development of novel, neuroprotective treatments. At this time, no such treatment is available [3], but the dramatic increase in our understanding of the molecular pathways involved in the pathogenesis of this disorder, and the availability of several animal models to perform preclinical testing of emerging therapeutic strategies, may nurture some hope for the future. The development of symptomatic treatment still plays an important role and there is a lack of good evidence from clinical trials. The boundaries between a HD specific treatment based upon knowledge of the complex pathogenic mechanisms involved at the molecular level and a causal treatment aimed at reversing some of these events may finally not be so sharp. In this review, some aspects of the complex molecular pathogenesis, for which our understanding has advanced so far, that clinical trials may be contemplated, will be examined. The status of clinical development of strategies based upon these mechanisms will be presented.

### Huntington’s Disease: a complex phenotype

The typical presentation of HD is the development of subtle cognitive and behavioural changes with involuntary, hyperkinetic, choreatic movements at around 40 years old. In the case of a dominant phenotype with a similar disorder in the family, a high accuracy diagnosis can be made, at least in those countries in Europe and America, where HD prevalence is relativly high. A number of other abnormal movements may be present, including dystonia, eye movement disturbances, bradykinesia, rigidity, gait and balance problems, myoclonus, and spasticity. Furthermore, they may have different presentations between young and old disease onset and evolve during the different stages of the disorder. Likewise, neuropsychological disturbances may be quite subtle at onset and evolve gradually over time, selectively affecting some aspects of cognition more than others. Difficulties in time assessment speed of cognitive processes and emotion detection may be detected early in the course of the disorder. They are typically followed by executive dysfunction, impairment in communication, memory and symptom unawareness [4]. Depressed mood, anxiety, irritability and apathy, are among the most frequently observed psychiatric signs [5], followed by obsessive compulsive symptoms and psychosis. They are also dependent upon the stage of the disorder. It is important to note, that there are substantial variations in this presentation, and some very early cases [6] have a developmental brain disorder, while late cases may be undistinguishable from senile chorea. This variation already stresses the importance of tailored treatment, as it is the case for symptomatic management [7].

Scales have been introduced to reliably assess the different aspects of the phenotype. The most frequent one is the United Huntington’s Disease (UHDRS) rating scale [8]. It comprises motor, cognitive, behavioural, and functional scales and has the advantage to offer precise instructions and training opportunities. The UHDRS may be complemented with additional specific instruments in order to assess particular aspects of the presentation.

When disease-modifying treatment are examined, two aspects need particular attention, the age at onset and the choice of biomarkers to assess progression. Age at onset is often taken as the time, when motor symptoms and signs occur. This is easily understandable, since these can be quite accurately seen and measured. However, subtle cognitive and psychiatric changes may already impair function, and might represent targets for treatment aiming at delaying disease onset. For this reason, Registry, the large observational study, run by the European Huntington’s Disease Network (EHDN), uses a form capturing the history of motor, behaviour (depression, irritability, apathy, aggressive behaviour, obsession, psychosis) and cognitive impairment in an open and unbiased way.

Furthermore, our increased understanding of the phenotype and the course of the disorders, including the development of biomarkers, allows the preparation of improved strategies in implementing therapeutic trials. The choice of biomarkers for protective studies will have to be tailored to which aspect of the neurodegenerative disease process and of the consecutive neuroplastic adaptation needs to be approached. An intensive, 3 years study of a cohort of HD gene carriers in a premanifest and in an early stage of the disease has recently been published [9-11]. A test battery including clinical cognitive, quantitative motor, oculomotor and neuropsychiatric measures as well as imaging methods was used to search for appropriate markers, which would demonstrate a change over the period of investigation. In the cross-sectional report comparing the three groups at baseline, differences in brain-imaging data, some neurophysiological motor and oculomotor tasks assessments, as well as cognitive and neuropsychiatric scales were found [9]. However, only a smaller number of these variables were subject to change during the longitudinal observation, stressing the importance of test validation in a prospective way. These data overall allow the suggestion that MRI assessments, including whole-brain atrophy, ventricular expansion, caudate atrophy, putamen atrophy, and white-matter atrophy, are valuable biomarkers both in the presymptomatic and in the early manifest stages. In the first, cognitive test may be added, for example the symbol digit modality test, the Stroop word reading and the emotion recognition test [11]. A follow-up protocol to these important studies in now in place and additional data are expected in the near future. They will inform the protocol of future clinical trials aimed at disease modification. Other studies have examined other clinical, cognitive, and neuroimaging assessments, and several biochemical parameters have also been suggested [12], but there is a need of replication and assessment in a longitudinal way before they can be seen as established for future clinical trials.

### The genetic background of HD

HD is due to an elongation of a CAG triplet repeat in exon 1 of the huntingtin (IT15) gene located on chromosome 4p16.3 [13]. Alleles with less than 27 CAG are normal and those with more than 39 fully penetrant for the disease phenotype, with an inverse correlation between the number of repeats and the age at onset. Repeats between 36 and 39 are incompletely penetrant and those between 27 and 35 are not associated with disease but with a higher risk for the next generation [14].

The CAG repeat is translated in a glutamine stretch of the huntingtin protein, which leads to the accumulation of an abnormal protein. Huntingtin itself has a number of functions in the cell, specifically in striatal spiny neurons, which all can be disturbed by the mutated protein [15]. These include modulation of gene transcription, impairment of protein trafficking and autophagy, disturbed energy metabolism and mitochondrial function, modulation of dynamic axonal transport mechanisms due to impaired microtubular dynamics, endocytic and vesicular trafficking changes at the presynaptic sites and modulation of postsynaptic signalling mechanisms. All these molecular events may theoretically be modulated by targeted pharmacological interventions. Some are more advanced along preclinical development paths and they are briefly reviewed here.

### Gene therapy

Strategies to inhibit the expression of the protein by the larger allele have been developed. They include different types of RNA molecules and antisense oligonucleotides (ASO) targeting the mutated transcript. Both strategies lead to the inhibition of HTT expression. This decrease in HTT expression improves the symptoms and prolongs survival in HD mouse models [16,17]. Problems, however, may occur in decreasing huntingtin overall expression, including the normal allele, since some cellular functions modulated by this protein may be impaired. In order to decrease expression only of the larger allele, strategies using sequences that specifically recognise SNP associated with the triplet repeat elongation to specifically target the mutated transcript are being explored [18].

Small interfering RNA (siRNA) may decrease mutated HTT RNA with a consecutive decrease in mutated protein and have been found to improve phenotype in animal models [19]. It is possible to design siRNA targeted at the mutant allele in a selective way allowing a normal expression of wild type huntingtin[20]. Mesenchymal stromal cells have been develop to express siRNA and tested with success in a number of animal models and their potential to treat HD is promising [21]. A trial using a construct with siRNA in healthy volunteers inoculated with respiratory syncytial virus has demonstrated its antiviral activity, thus establishing a proof of concept for such an approach [22]. Of course the challenge of delivering siRNA in the brain is higher than in the readily accessible upper airways and the issue of long-term safety remains a concern, although the stereotactic intraparenchymal delivery of siRNA packaged into a modified andenovirus has been found safe for 6 months in primates [23]. In treated animals, HTT mRNA levels fell by 28% compared with control animals receiving unspecific RNA, protein levels also significantly decreased. A programme had been announced to start phase I studies with continuous intracerebral application of an RNAi therapeutic by using a pump system [24], however this has been retracted again, showing the difficult path of development for such strategies. Chemical modifications of siRNA may improve stability with retaining high silencing capability and tissue spread after local application [25].

Ansisense oligonucleotide (ASO) may be prepared to target specifically HTT mRNA [26], inducing their degradation by RNase activity. They can be modified to make them resistant to exonuclease cleavage, which improve their stability. After injection in the mammalian brain, they decrease HTT mRNA levels in the striatum without any morphological change. There a time lag in the restoration of mutant HTT mRNA levels there after transient inhibition [27]. A clinical trial to examine safety, tolerability and activity of an ASO treatment is presently undergoing in amyotrophic lateral sclerosis caused by SOD1 gene mutations (http://clinicaltrials.gov/ identifier: NCT01041222).

Challenges concerning HTT gene silencing remain, they include the delivery process and target volume choice, timing of treatment along the course of the disease, as well as long-term effects and side effects, but these strategies offer promise for a real impact in changing the course of the disorder in a meaningful way.

### Targeting the mutated protein

The CAG repeat expansion is translated in a glutamine stretch of the huntingtin molecule, a protein, which undergoes a number of posttranslational modifications, including acetylation, phosphorylation, SUMOylation, ubiquitination, palmytoilation and proteolytic cleavage. This has lead to the suggestion, that modification of these posttranslational processes might modulate the levels of abnormal huntingtin [28].

The polyphenol (2)-epigallocatechin-3-gallate (EGCG), a component of green tee, inhibits the aggregation of mutated huntingtin in vitro [29], the starting of a phase II trial in HD has been reported (http://clinicaltrials.gov, identifyer: NCT01357681).

Sirtuin 1 is involved in the postranslational modification of Huntingtin and acts by deacetylation of the protein. Sirtuin 1 inhibition has been postulated to lead to increase clearance of mutated huntingtin (http://www.paddingtonproject.eu/). A randomised, double-blind placebo-controlle phase iB pharmacodynamic study has now been run in HD patients [30].

Another way would be to develop strategies using chaperones to improve clearance of the abnormal protein. Indeed removal of the abnormal protein, for example by over expression of a human heat shock protein in HSJ1a in mice bearing the HD mutation, lead to a significant decrease in insoluble HTT and size of inclusions [31]. Another way to clear the abnormal protein in order to decrease its toxic effects would be to engineer antibody specifically reacting with mutated huntingtin. Single-chain, single-domain antibodies targeted to intracellular huntingtin with expanded glutamine track have been used in several animal models with improvement of the phenotype [32].

#### Improving mitochondrial function

Mitochondrial function is impaired in HD as demonstrated by a decrease in the function of several complexes along the electron transport chain [33,34], decrease in mitochondrial density, and morphological alteration in brains from HD patients [35]. Mutated huntingtin impairs mitochondrial function by a number of different mechanisms, including permeability transition pore modulation, decrease of PGCα expression, stimulation of glutamate receptor by increased depolarisation, mitochondrial fractionation, and impaired respiratory chain function [36]. Several therapeutic approaches have been followed based on this background.

Coenzyme Q, a lipid-soluble molecule with antioxidant, and membrane-stabilizing properties, is involved in oxidative phosphorylation and represent a potential neuroprotective compound suggested to improve mitochondrial function. Coenzyme Q has been tested in HD, however, only a trend in improvement was measured. A follow-up trial has tested higher doses, and they appear to be safe [37]. There are currently two studies, sponsored by the National Institutes of Health in the USA further exploring the effect of Coenzyme Q10 in patients with HD. One is a multicenter phase II, randomized double blind controlled trial to test the tolerability of high doses (600 to 2400 mg per day) in pre-manifest participants with CAG triplet elongation (http://clinicaltrials.gov, identifier: NCT00920699). The other is a long-term randomized double blind study to examine whether coenzyme Q10 is effective in slowing HD symptom worsening. Almost 50 sites are involved for this ongoing, 5 years-long trial, for which enrolment has been closed (http://clinicaltrials.gov, identifier: NCT00608881).

Creatine is a high-energy phosphate donor, which has been tested in animal models of HD. In patients with HD 8g/day of creatine was well-tolerated and improved a biochemical indicator of oxidative injury [38]. Another, multicentre study with a long-term perspective of 3 years to examine the effect of high-dose creatine on progression of functional decline in adults with early clinical signs of HD is still on-going (http://clinicaltrials.gov, accession number NCT00712426).

#### Cell repair strategies

Several strategies have been explored to replace degenerated neurons in order to restore networks in the striatum. In earlier pilot studies, bilateral transplantation of embryonic tissue in the caudate of HD patients has lead to a short benefit in some patients [39]. A long-term multicentric study on the efficacy of intracerebral grafting is now ongoing in Europe and results are expected after final completion scheduled in Mai 2013 (http://clinicaltrials.gov, identification number: NCT00190450). One short-coming is the fact, that neuronal transplants undergo a disease-related degeneration similar to the host [40]. Immune response with rejection of the graft may also be an important limiting factor [41]. Ethical issues, the poor availability and inhomogeneity of donor tissues represent other problems linked with the use of fresh embryonic tissue. For these reasons, other strategies for cell replacement in HD have been followed.

Human embryonic stem cells have been developed and shown to improve clinical phenotype in animal models of HD, however, their use in human may be hampered by immunological and trophic mechanisms [42]. Somatic cells may be reprogrammed into pluripotent stem cells, which would provide a source of cells for replacement therapy. In a recent landmark study, the mutation could be corrected in pluripotent stem cells derived fibroblasts from patients with HD by the replacement of the expanded CAG repeat with a normal repeat. These cells developed into neurons in vitro and in vivo, which had a normalised molecular phenotype [43]. Such a strategy may open new venues in cell therapy for the treatment of HD patients in later stages of the disorder, when other means of treatment are not possible anymore.

Finally, cells may also be reengineered to produce trophic factors [42]. For example astrocytes may be induced to producing BDNF, and have demonstrated some encouraging success in animal studies [44]. Novel technologies allow the preparation of mesenchymal stromal cells to treat a number of disorders and phase I-III studies are underway, for example to treat chronic liver disease [45] or multiple sclerosis [46].

#### The place of symptomatic therapies

The implementation even of the most promising strategies aimed at modifying the disease process, in order to delay onset and slow progression will take years at least. There is therefore the need to improve on symptomatic treatments, which can be classified in two overlapping categories. A specific approach is based on knowledge of the disease molecular mechanisms or on studies examining an aspect of the phenotype in a HD. A non-specific approach, which has been mostly used in the past, is based on the phenotypical characterisation and generalisation from other disorders, in which aspects of the phenotype are shared. Unfortunately, only few high quality clinical trials have been performed specifically for treatment of symptoms in HD patients [47]. We have performed a survey of the treatment of HD chorea in different regions and found important differences, pointing at the lack of consensus [48]. Needs of the patient, including stigma, physical injury, gait instability, work interference, and disturbed sleep were indications to start treatment of chorea. However, the choice of drug varied substantially.

Tetrabenazine is the only drug approved by the USA food and drug administration for the treatment of chorea in HD [49]. The major mechanism of tetrabenazine action is the inhibition of a vesicular monoamine transporter (VMAT). Brain VMAT2 is active in cytoplasmatic dopamine transport and storage in synaptic vesicles. In a short-term randomised control trial involving 84 patients over 12 weeks, Tetrabenazine, at adjusted doses up to 100 mg per day, was shown to decrease the UHDRS chorea subscore by 5 points (placebo 1.5) [50]. There was also an improvement in the global clinical impression. After discontinuation of the drug, chorea worsened again. In a second, smaller randomized controlled trial of tetrabenazine withdrawal worsening of 5 points was found [51]. One recent study has been devoted to the treatment of cognitive impairment specifically in HD.

In a double blind assessment did treatment with latrepirdine for 6 months not improve cognition or function relative to placebo [52]. Deep brain stimulation has also been suggested on the basis of an understanding of basal ganglia dysfunction in HD to treat chorea [53]. There is certainly a need for future trials on drugs and other non-pharmacological treatment based on improved knowledge of the pathophysiology of the disorder.

## Conclusion

As shown already be the few examples cited above, the field of translational research in HD is moving at a fast speed and hope for breakthrough in several areas can now be nurtured in a cautious way. At the present time, several avenues are quite promising, they include gene silencing strategies to decrease expression of the transcript with elongated triplet repeat part, Huntingtin modifying compounds, cell strategies to provide trophic factors, and molecules targeting mitochondria. Several other pathways are expected to emerge further as well. In any case, the complexity of the pathophysiological mechanisms involved in HD will be reflected by a complex set of therapies with synergistic properties, and it is not to be expected that a single treatment would be sufficient.

The recognition that HD, due to the fact that the cause is precisely known and can be recognised long before onset, may be a paradigm for disease modifying treatment in neurodegenerative disorders in general, has fostered interest of researchers, pharmaceutical companies and sponsors alike. The involvement of the clinical care community and, of course, of the people affected themselves, together with above partners, is being highly instrumental for the progress in this field. This has been well exemplified by the work of the Huntington Study Group, the EHDN, and will certainly be the case as well by newly established networks like the Latin American HD Network, and the Chinese Huntington’s Disease Network.

## Competing interests

The author declare that he has no competing interests.
